# Propofol reduces breast cancer cell stemness via FOXO3/SOX2 axis

**DOI:** 10.7150/jca.104142

**Published:** 2025-01-27

**Authors:** Wen-Qian Fang, Xiao-Bei Zhang, Yue Yu, Jie Ge, Ran Meng

**Affiliations:** 1 Department of Anesthesiology, Tianjin Central Hospital of Gynecology and Obstetrics, Tianjin Key Laboratory of Human Development and Reproductive Regulation, Nankai University Affiliated Hospital of Gynecology and Obstetrics, Tianjin 300100, China.; 2 the First Department of Breast Cancer, Tianjin Medical University Cancer Institute and Hospital, National Clinical Research Center for Cancer, Tianjin 300060, China.; 3 Key Laboratory of Breast Cancer Prevention and Therapy, Tianjin Medical University, Ministry of Education, Tianjin 300060, China.; 4 Key Laboratory of Cancer Prevention and Therapy, Tianjin 300060, China.; 5 Tianjin's Clinical Research Center for Cancer, Tianjin 300060, China.

**Keywords:** propofol, breast cancer, cancer stem cells, FOXO3

## Abstract

**Objective:** Propofol is a common intravenous anesthetic in cancer resection surgery, which is considered to exhibit anti-tumor effect in various cancer types. This study was aimed at investigating the role and mechanism of propofol in breast cancer stemness and proliferation.

**Methods:** The breast cancer cells with propofol treatment were sequenced. The expression of FOXO3 in propofol treated cells was detected by RT-qPCR and Western blot. The CSC properties were analyzed by screen cells with ESA^+^CD44^+^CD24^-/low^ through flow cytometry and the proliferation capacity were also detected. The expression correlation of FOXO3 and target genes were detected by western blot. The potential binding site of FOXO3 on SOX2 was predicted by JASPAR and verified by dual-luciferase reporter assay and ChIP assay.

**Results:** FOXO3 was found to be upregulated in propofol 24h-treated cells. Propofol could inhibit the capacity of breast cancer cell stemness and proliferation by upregulation FOXO3, which inhibited SOX2 expression transcriptionally.

**Conclusion:** In this study, we uncovered the role of propofol-FOXO3-SOX2 in breast cancer cell stemness and proliferation, which might serve as potential targets for breast cancer therapy.

## Introduction

Breast cancer is the most common malignancy that occurs among women worldwide^1^, ^2^. Breast cancer is considered as a multifactorial disease, and recently, accumulating evidence have demonstrated the crucial roles of specific genes, RNAs and drugs in the treatment of breast cancer^3^-^5^. Different types of cancer cells have been reported to perform different roles in cancer progression^6^, among which, cancer stem cells (CSCs) mainly participate in therapeutic resistance and reinitiate cancer with all its heterogeneity^7^. So far, numerous efforts have been made to characterize and eradicate breast cancer stem cells (BCSCs), providing emerging strategies for breast cancer therapy.

Propofol (2,6-diisopropylphenol) is a kind of intravenous anesthetic, which is commonly employed in cancer resection surgery. Propofol, dubbed as “milk of anesthesia due to its white color, is characterized by rapid induction and quick recovery. The anti-cancer effect of propofol has been reported in recent studies, at the meanwhile, the potential mechanisms have attracted increasing attention. Propofol is reported to be involved in many biologic processes and can interact with different elements. It has been reported that propofol dramatically up-regulated miR-199a expression and inhibited the invasion of HepG2 cells via the downregulation of matrix metalloproteinase-9 (MMP-9)^8^. Wang *et al.* also reported the tumor suppression effect of propofol by up-regulation of miR-328 in pancreatic cell line proliferation, and invasion^9^. Besides, propofol is also demonstrated to be involved in multiple cancer related pathways. In pancreatic cancer, propofol was found to inactivate NF-κB signaling and lead to gemcitabine chemosensitization and cell apoptosis^10^. In breast cancer, a recent study reported that propofol significantly inhibited breast cancer cell proliferation and augmented the anti-tumor effects of paclitaxel and doxorubicin by promoting ferroptosis^11^. Zhang *et al.* demonstrated that propofol can decrease the mammosphere formation of breast cancer stem cells through PDL1/Nanog pathway^12^. However, the role and mechanism of propofol in breast cancer progression still needs further investigation.

In our study, the gene expression profiles of breast cancer cells treated with propofol for 6h and 24h were analyzed. FOXO3 (Forkhead Box O3) was identified to be up regulated in 24h propofol treatment breast cancer cells. Consistent with this, previous studies have reported the upregulation of FOXO3 level after propofol treatment^13^, ^14^. FOXO3a belongs to the subfamily of forkhead transcription factors and perform essential characters in the basic cellular biological processes, including apoptosis, proliferation, cell cycle regulation, and DNA damage^15^. Previous studies identified FOXO3 as a tumor suppressor, which is downregulated in cancer cells and tissues^16^, ^17^. Yao *et al.* reported that in the mitochondrial dysfunction of hepatocellular carcinoma, CDK9 was able to enhance the therapeutic effects by regulating SIRT1-FOXO3-BNIP3 axis and involved in mitophagy mediated by PINK1-PRKN^18^. Besides, FOXO3a can be hypermethylated by DNA (cytosine-5-)-methyltransferase 1 (DNMT1) and resulted in its downregulation, which led to the promotion of breast cancer stem cell properties and tumorigenesis^19^. While the further mechanism of FOXO3 in BCSCs regulation remains unclear.

Herein, we investigated the role of propofol and explored the mechanism of propofol-FOXO3 in BCSCs and breast cancer proliferation.

## Material and Methods

### Cell culture

The breast cancer cell lines MCF7 and MDA-MB-231 cells, as well as human embryonic kidney cells 293FT were obtained from the Cell Bank of the Chinese Academy of Sciences (Shanghai, China). The cells were cultured at 37℃ in 5% CO_2_, as previously described. Propofol was dissolved in 10% intralipid (AstraZeneca) and the mammosphere cells of MCF7 and MDA-MB-231 were grown in 6-well plates and treated with 10 μm propofol for 48 h. For transient transfection, plasmids or siRNAs were transfected into different cell lines using FuGENE HD Transfection Reagent (Promega, Madison, WI, USA).

### Cmap database

Gene expression profiles of MCF7 cell lines treated with propofol for 6 or 24 hours were downloaded from the Cmap database (https://clue.io/), containing Z-score normalized expression profiles of 12328 genes. Of these, the expression of 978 landmark genes was determined directly, and the expression of the remaining genes was inferred from the expression of landmark genes according to the algorithm of the database.

### RNA extraction and RT-qPCR

Total RNA was isolated from cells using the TRIzol Reagent (Life Technologies) according to the standard protocol. The mRNA expression was determined using the GoTaq qPCR Master Mix (Promega). Gene expression fold changes were assessed using the 2^-ΔΔCt^ method. GAPDH was used as the endogenous control. PCR primers used are listed as follows:

FOXO3: Forward: 5'-AGTGGATGGTGCGCTGTGT-3'; Reverse: 5'-CTGTGCAGGGACAGGTTGT-3'.

SOX2: Forward: 5'-GCTCGCAGACCTACATGAAC-3'; Reverse: 5'-GGGAGGAAGAGGTAACCACA-3'.

GAPDH: Forward: 5'-CAAGGTCATCCATGACAACTTTG-3'; Reverse: 5'-GTCCACCACCCTGTTGCTGTAG-3'.

### Western blot

The cells were lysed with Radio Immunoprecipitation Assay (RIPA) lysis buffer containing 1 mM phenylmethanesulfonyl fluoride (PMSF) (Solarbio, Beijing, China). Bicinchoninic Acid Assay (BCA) Protein Assay Kit (Thermo Fisher Scientific, San Jose, CA, USA) was used to determine protein concentration. Protein samples were boiled, separated on 8-10% SDS-PAGE, and then transferred to polyvinylidene fluoride (PVDF) membranes (Millipore, Bedford, MA, USA). The membranes were blocked for 1 h with 5% (w/v) skimmed milk at room temperature and incubated with primary antibody (Nanog: Abcam ab109250, OCT-4: cell signaling #2750, SOX2: cell signaling #23064, FOXO3: Abcam ab109629 and β-actin: cell signaling #4967) at 4℃ overnight. After washing with Tris Buffered Saline with Tween (TBST) three times, the membranes were incubated with horseradish peroxidase (HRP)-conjugated secondary antibody for 1 h at room temperature. Enhanced chemiluminescence (ECL) reagent (Millipore) was used to visualize the blots.

### Chromatin immunoprecipitation

ChIP-qPCR analysis was performed by the manufacturer's recommendations (Millipore) or with an isotype control as previously described^20^. The immunoprecipitated DNA was analyzed using agarose gel electrophoresis with primers specific to the SOX2 promoter region (5'-GCGTCCCATCCTCATTTAAGT-3' and 5'-TCCTCCACTCGAGCCCAGCCT-3').

### Dual-luciferase reporter assay

The luciferase activities were determined using the Luciferase assay system (Promega) according to the instructions. 5×10^4^ 293FT cells were seeded in 12-well plates. The cells were transfected with 200 ng of the indicated firefly luciferase reporter plasmid, 200 ng of the expression plasmid and 20 ng of Renilla reporter using FuGENE HD for 48 h. pRL-TK Renilla reporter was used as a normalization control.

### 3-(4,5-dimethylthiazol-2-yl)-2,5-diphenyltetrazolium bromide (MTT)

2×10^3^ cells were seeded in 96-well plates after transfection for 48 h. Each well was incubated with 10 μL MTT for 4 h. The medium was discarded, and the formazan produced after MTT treatment was dissolved in 150 μL DMSO. The absorbance was measured at 570 nm using a micro-plate reader (Bio-Rad, Richmond, CA, USA). The cell viability was measured at the indicated times.

### Flow cytometry

The cells were digested and fully dispersed into a single cell solution and then were labeled with ESA-FITC, CD44-APC, and CD24-PE antibodies as previously described^21^. The proportion of ESA^+^/CD44^+^/CD24^-/low^ cells was tested by flow cytometry.

### Statistical analysis

SPSS 24.0 (IBM, Armonk) was used for data analysis. All measurement data were exhibited as mean ± standard deviation. A one-way ANOVA or Student's t-test was used to determine group differences. P-value < 0.05 was considered statistically significant.

## Results

### The expression FOXO3 is up-regulated after propofol treatment in breast cancer

**Figure 1:**
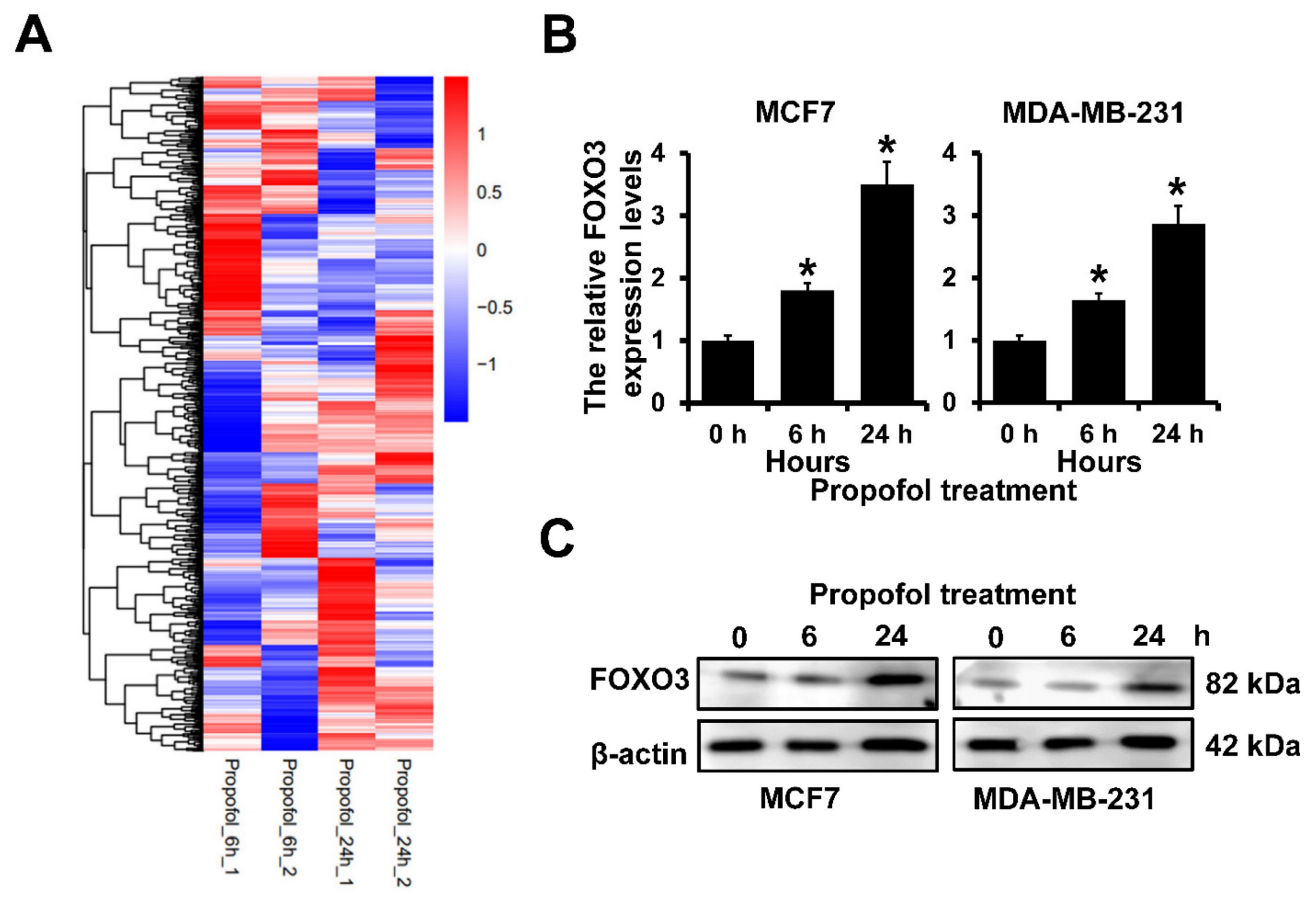
** The expression of FOXO3 in breast cancer after propofol treatment. A,** The differentially expressed gene in MCF7 cells after propofol treatment for 24 hours from Cmap database. **B,** RT-qPCR analysis of FOXO3 mRNA expression in MCF7 (left) and MDA-MB-231 (right) after propofol treatment for indicated hours. **C,** Western blot analysis of FOXO3 protein expression in MCF7 (left) and MDA-MB-231 (right) after propofol treatment for indicated hours. *P < 0.05.

Our previous study indicated that mammosphere culture enriches breast cancer stem cells and propofol could reduce the mammosphere formation of breast cancer^21^. However, the mechanism is still uncovered. To address this, we analyzed the differentially expressed genes in MCF7 cells after treatment with propofol for 6 and 24 h. We observed that 978 landmark genes were changed after being treated with propofol for 6 h and 24 h. The expression of FOXO3 was significantly increased in MCF7 cells after treatment with propofol for 24 h (Figure 1A). Furthermore, RT-qPCR and western blot analyses indicated that propofol treatment could elevate both the mRNA and protein expression of FOXO3 in a dose dependent manner (Figure 1B and 1C). Together, these results indicated that propofol can induce the FOXO3 expression.

### Propofol reduces the mammosphere formation of breast cancer by FOXO3

**Figure 2:**
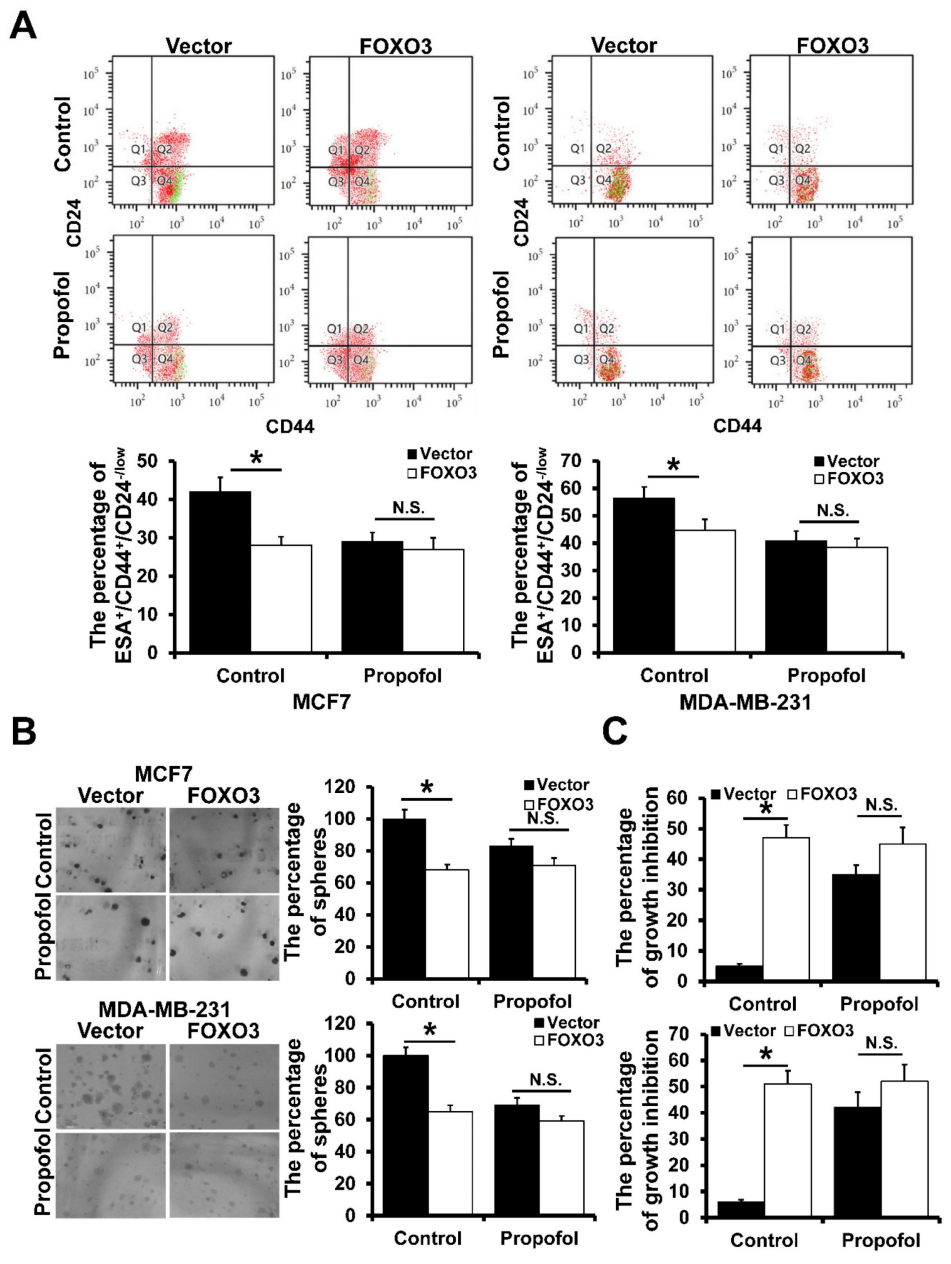
** The effect of FOXO3 on breast cancer cell stemness. A,** The effect of FOXO3 on the percentage of ESA^+^CD44^+^CD24^-/low^ in mammosphere of breast cancer cell lines with or without propofol treatment. **B,** The effect of FOXO3 on the mammosphere-forming ability of breast cancer cell lines with or without propofol. **C,** The effect of FOXO3 on the proliferation ability of BCSCs with or without propofol. *P < 0.05.

To investigate the effect of propofol and FOXO3 on breast cancer cell stemness, we analyzed the percentage of ESA^+^/CD44^+^/CD24^-/low^ cells by flow cytometry. Propofol could significantly reduce the percentage of ESA^+^/CD44^+^/CD24^-/low^ cells, whereas there was no significant difference between the propofol and control group in FOXO3-overexpressed MCF7 and MDA-MB-231 cells (Figure 2A). Furthermore, propofol could significantly inhibit mammosphere formation ability in both MCF7 and MDA-MB-231 stem cells, and FOXO3 overexpression could eliminate this phenomenon (Figure 2B). In addition, propofol had no effect on breast cancer stem cell proliferation after FOXO3 overexpression (Figure 2C). Taken together, these results suggested that propofol reduced the mammosphere formation by up-regulation of FOXO3.

### FOXO3 transcriptionally inhibited SOX2 expression

**Figure 3:**
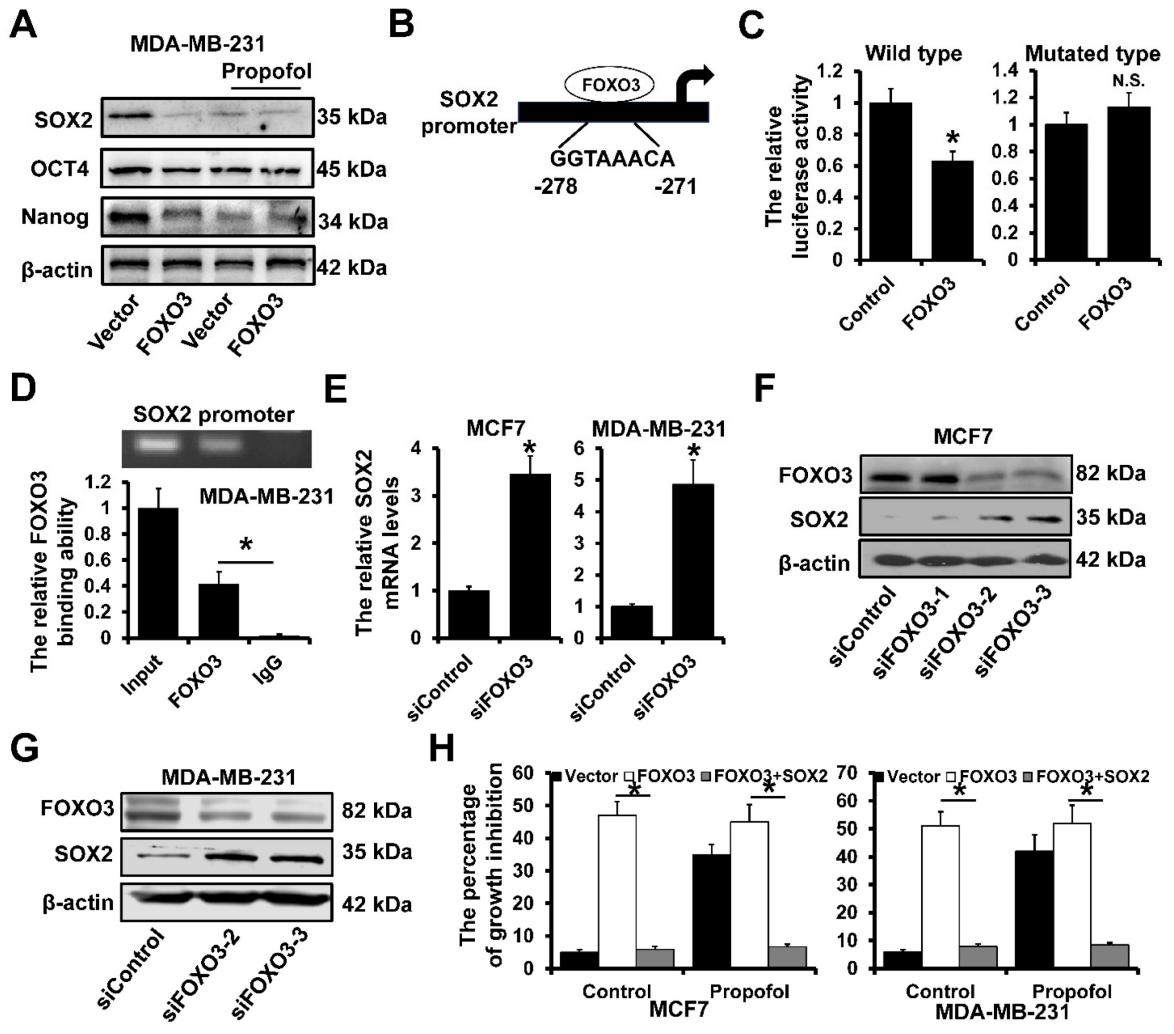
** FOXO3 transcriptionally represses SOX2 expression. A,** The expression of stem cell-associated proteins SOX2, OCT4 and Nanog in FOXO3-overexpressed MDA-MB-231 and control cells with or without propofol treatment for 24 hours by western blot. **B,** A potential FOXO3‑binding sites located in the SOX2 promoter region. **C,** Dual-luciferase reporter assays were used to analyze the regulation of SOX2 promoter activity by FOXO3. **D,** The interaction between FOXO3 and SOX2 promoter region in indicated cells was verified using ChIP analysis. **E,** The effects of FOXO3 on SOX2 expression were determined using RT-qPCR.** F** and **G**, The effects of FOXO3 on SOX2 expression were determined using western blot in MCF7 cells (F) and MDA-MB-231 cells (G). H, The percentages of growth inhibition of FOXO3-upregulated and SOX2-upregulated MCF7 cells (left) and MDA-MB-231 cells (right). *P < 0.05.

We next determine the effect of propofol and FOXO3 on the expression of the stem cell-associated proteins. As shown in Figure 3A, the expression of SOX2, OCT4 and Nanog was significantly decreased in MDA-MB-231 cells after treatment with propofol. However, there was no effect of propofol on the expression of SOX2, OCT4 and Nanog in FOXO3-overxpressed MDA-MB-231 cells. Further analysis by JASPAR website (https://jaspar.genereg.net/) of the SOX2 promoter sequence (-1000 ~ +1 to TSS) revealed a potential FOXO3 binding sites (Figure 3B). We next cloned the SOX2 promoter into the pGL3-basic reporter and transfected the reporters into 293FT cells and performed luciferase assays to measure promoter activity. The FOXO3 overexpression significantly decreased the luciferase activity of the constructs but not that of the mutated construct (Figure 3C). Moreover, ChIP demonstrated that FOXO3 could bind to the SOX2 promoter region in the MDA-MB-231 cells (Figure 3D). Then, the FOXO3 siRNAs were utilized to knock down the expression of FOXO3, which included siFOXO3-1, siFOXO3-2 and siFOXO3-3. These three siRNAs were mixed and transfected into MCF7 and MDA-MB-231 cells for better efficiency. The RT-qPCR results showed that SOX2 mRNAs were dramatically up-regulated in siFOXO3 cells (Figure 3E). For further verifying the regulation of FOXO3 on SOX2 proteins, the western blot assay was performed in MCF7 cells, which resulted that downregulation of FOXO3 led to an increase of SOX2. Besides, among three siRNAs of FOXO3, siFOXO3-2 and siFOXO3-3 showed better efficiencies (Figure 3F). Then, the siFOXO3-2 and siFOXO3-3 were transfected into MDA-MB-231 cells and exhibited the same results (Figure 3G). In addition, in order to explore whether the inhibition of FOXO3 on cell proliferation was realized by regulating SOX2 under the treatment of propofol, the SOX2 was overexpressed in FOXO3-upregulated cells. The results showed that propofol treatment increased the percentage of growth inhibition by regulating FOXO3 expression, while when SOX2 was upregulated, the growth inhibition effect was cancelled out (Figure 3H). These results indicated that the propofol induced FOXO3-upregulation can inhibit breast cancer cell stemness by transcriptionally inhibiting SOX2 expression.

## Discussion

Numerous studies have demonstrated the regulation of propofol on CSCs. CSCs can differentiate into different types of tumor cells and also exhibit a long-term self-renewal capability, which contributes to its extremely important role in the development and occurrence of malignant tumors, including breast cancer^22^-^24^. Rephael *et al.* reported that Propofol exerted a dose-dependent inhibitory effect on the self-renewal, expression of mesenchymal markers, and migration of glioma stem cells via BDNF-AS and extracellular vesicles^25^. In bladder cancer, propofol was reported to inhibit cancer cell proliferation and stem-like properties by inhibiting hedgehog pathway^26^. Besides, a latest study illustrated that propofol was able to suppress colon cancer cell stemness and EMT process by regulating STRT1, Wnt signaling and AKT signaling^27^. Compared with other breast cancer cell lines, BCSCs exhibited a high survival rate under the treatment of chemotherapy drugs^28^. Therefore, CSCs are considered as a malignant phenotype, and effective removal of CSCs is crucial for achieving the expected anticancer efficacy.

Our previous study identified that ESA^+^CD44^+^CD24^-/low^ breast cancer cells have stem-like cell characteristics^29^-^31^. In our present study, we found that the stemness of breast cancer cells was significantly inhibited by overexpressed FOXO3 after propofol treatment. FOXO3 has been reported to regulate cancer cell stemness in previous studies. For example, An *et al.* demonstrated that SIRT1 could inhibit gastric cancer stemness by activating AMPK/FOXO3 positive feedback loop^32^. Kumazoe *et al.* reported that FOXO3/PGC-1β pathway was crucial for CSC properties of pancreatic ductal adenocarcinoma^33^. Specifically, FOXO3 is associated with CD44 expression in multiple cancer cells, which is a major CSC marker^34^, ^35^. In breast cancer, the downregulation of FOXO3a by DNMT1 could promote breast cancer stem cell properties and tumorigenesis^36^. However, more detailly mechanisms are unclear.

SOX2 (sex-determining region Y-box 2) is a well-characterized pluripotent factor, and has been identified as an anti-cancer target, which is crucial for stem cell self-renewal, reprogramming, and homeostasis^37^. Zhu *et al.* illustrated that SOX2 promoted colorectal cancer stem cells properties, and EMT process by regulating β-catenin and Beclin1/autophagy signaling^38^. Sepideh *et al.* reported that SOX2 has capacity of increasing growth and metastasis of cancer cells, besides, SOX2 could also promote stemness of tumor cells and increases the number of CSCs^39^. The regulation of FOXO3 on SOX2 has been reported in previous studies. In head and neck squamous cell carcinoma, Yang *et al.* found that autophagy could regulate cancer stem cell phenotype via noncanonical FOXO3/SOX2 axis^40^. Specifically, FOXO3 can bind with the promoter region of SOX2 and inhibit its expression^41^. While in breast cancer, the regulation of FOXO3 on SOX2 has also been reported^36^, but still need further investigation.

In our current study, we found that in breast cancer cells, overexpression of FOXO3 also downregulated the expression of SOX2. Besides, the combination of FOXO3 and SOX2 promoter was verified by dual-luciferase reporter assay and ChIP assay. It's our study come up with that propofol could inhibit cancer cell stemness and proliferation by regulation of FOXO3/SOX2 axis firstly. These findings might bring new sights into breast cancer treatment strategies and these elements are expected to be potential targets for breast cancer therapy.

## References

[1] Veronesi U, Boyle P, Goldhirsch A, Orecchia R, Viale G (2005). Breast cancer. Lancet.

[2] Siegel RL, Miller KD, Wagle NS, Jemal A (2023). Cancer statistics, 2023. CA Cancer J Clin.

[3] Barzaman K, Karami J, Zarei Z (2020). Breast cancer: Biology, biomarkers, and treatments. Int Immunopharmacol.

[4] Tian Y, Liu X, Hu J (2021). Integrated Bioinformatic Analysis of the Expression and Prognosis of Caveolae-Related Genes in Human Breast Cancer. Front Oncol.

[5] Zhu KY, Tian Y, Li YX (2022). The functions and prognostic value of Kruppel-like factors in breast cancer. Cancer Cell Int.

[6] Hutchinson L (2010). Breast cancer: challenges, controversies, breakthroughs. Nat Rev Clin Oncol.

[7] Zeng X, Liu C, Yao J (2021). Breast cancer stem cells, heterogeneity, targeting therapies and therapeutic implications. Pharmacol Res.

[8] Zhang J, Zhang D, Wu GQ, Feng ZY, Zhu SM (2013). Propofol inhibits the adhesion of hepatocellular carcinoma cells by upregulating microRNA-199a and downregulating MMP-9 expression. Hepatobiliary Pancreat Dis Int.

[9] Yu X, Gao Y, Zhang F (2019). Propofol inhibits pancreatic cancer proliferation and metastasis by up-regulating miR-328 and down-regulating ADAM8. Basic Clin Pharmacol Toxicol.

[10] Du QH, Xu YB, Zhang MY, Yun P, He CY (2013). Propofol induces apoptosis and increases gemcitabine sensitivity in pancreatic cancer cells in vitro by inhibition of nuclear factor-kappaB activity. World J Gastroenterol.

[11] Sun C, Liu P, Pei L, Zhao M, Huang Y (2022). Propofol Inhibits Proliferation and Augments the Anti-Tumor Effect of Doxorubicin and Paclitaxel Partly Through Promoting Ferroptosis in Triple-Negative Breast Cancer Cells. Front Oncol.

[12] Zhang X, Li F, Zheng Y (2019). Propofol Reduced Mammosphere Formation of Breast Cancer Stem Cells via PD-L1/Nanog In Vitro. Oxid Med Cell Longev.

[13] Wang H, Zhang S, Zhang A, Yan C (2018). Propofol Prevents the Progression of Malignant Pheochromocytoma In Vitro and In Vivo. DNA Cell Biol.

[14] Feng S, Sun Y (2018). Protective role of propofol in endometriosis and its mechanism. Exp Ther Med.

[15] Calissi G, Lam EW, Link W (2021). Therapeutic strategies targeting FOXO transcription factors. Nat Rev Drug Discov.

[16] Habrowska-Gorczynska DE, Koziel MJ, Kowalska K, Piastowska-Ciesielska AW (2021). FOXO3a and Its Regulators in Prostate Cancer. Int J Mol Sci.

[17] Liu Y, Ao X, Ding W (2018). Critical role of FOXO3a in carcinogenesis. Mol Cancer.

[18] Yao J, Wang J, Xu Y (2022). CDK9 inhibition blocks the initiation of PINK1-PRKN-mediated mitophagy by regulating the SIRT1-FOXO3-BNIP3 axis and enhances the therapeutic effects involving mitochondrial dysfunction in hepatocellular carcinoma. Autophagy.

[19] Liu H, Song Y, Qiu H (2020). Downregulation of FOXO3a by DNMT1 promotes breast cancer stem cell properties and tumorigenesis. Cell Death Differ.

[20] Chen ZH, Chen YB, Yue HR (2023). PAX5-miR-142 feedback loop promotes breast cancer proliferation by regulating DNMT1 and ZEB1. Mol Med.

[21] Liu BW, Sun N, Lin H, Zhou XJ, Ma HY, Wang X, Cao XC, Yu Y (2023). The p53/ZEB1-PLD3 feedback loop regulates cell proliferation in breast cancer. Cell Death Dis.

[22] Bell DR, Van Zant G (2004). Stem cells, aging, and cancer: inevitabilities and outcomes. Oncogene.

[23] Shafee N, Smith CR, Wei S (2008). Cancer stem cells contribute to cisplatin resistance in Brca1/p53-mediated mouse mammary tumors. Cancer Res.

[24] Bai X, Ni J, Beretov J, Graham P, Li Y (2018). Cancer stem cell in breast cancer therapeutic resistance. Cancer Treat Rev.

[25] Nizar R, Cazacu S, Xiang C (2023). Propofol Inhibits Glioma Stem Cell Growth and Migration and Their Interaction with Microglia via BDNF-AS and Extracellular Vesicles. Cells.

[26] Li G, Zhang X, Guo X, Li Y, Li C (2021). Propofol Inhibits the Proliferation, Migration, and Stem-like Properties of Bladder Cancer Mainly by Suppressing the Hedgehog Pathway. Cell Transplant.

[27] Wang R, Li S, Hou Q (2023). Propofol inhibits colon cancer cell stemness and epithelial-mesenchymal transition by regulating SIRT1, Wnt/beta-catenin and PI3K/AKT/mTOR signaling pathways. Discov Oncol.

[28] Yu F, Yao H, Zhu P (2007). let-7 regulates self renewal and tumorigenicity of breast cancer cells. Cell.

[29] Zhang X, Zhang S, Liu Y (2012). Effects of the combination of RAD001 and docetaxel on breast cancer stem cells. Eur J Cancer.

[30] Liu Y, Zhang X, Liu J, Hou G, Zhang S, Zhang J (2014). Everolimus in combination with letrozole inhibit human breast cancer MCF-7/Aro stem cells via PI3K/mTOR pathway: an experimental study. Tumour Biol.

[31] Zhu Y, Zhang X, Liu Y (2012). Antitumor effect of the mTOR inhibitor everolimus in combination with trastuzumab on human breast cancer stem cells in vitro and in vivo. Tumour Biol.

[32] An Y, Wang B, Wang X, Dong G, Jia J, Yang Q (2020). SIRT1 inhibits chemoresistance and cancer stemness of gastric cancer by initiating an AMPK/FOXO3 positive feedback loop. Cell Death Dis.

[33] Kumazoe M, Takai M, Hiroi S (2017). The FOXO3/PGC-1beta signaling axis is essential for cancer stem cell properties of pancreatic ductal adenocarcinoma. J Biol Chem.

[34] Kumazoe M, Takai M, Bae J (2017). FOXO3 is essential for CD44 expression in pancreatic cancer cells. Oncogene.

[35] Kim EY, Lee SU, Kim YH (2022). 1,2,3,4,6-Penta-O-galloyl-beta-D-glucose Inhibits CD44v3, a cancer stem cell marker, by regulating its transcription factor, in human pancreatic cancer cell line. Anim Cells Syst (Seoul).

[36] Wang Q, Li C, Zhu Z, Teng Y, Che X, Wang Y, Ma Y, Wang Y, Zheng H, Liu Y, Qu X (2016). miR-155-5p antagonizes the apoptotic effect of bufalin in triple-negative breast cancer cells. Anticancer Drugs.

[37] Zhang S, Xiong X, Sun Y (2020). Functional characterization of SOX2 as an anticancer target. Signal Transduct Target Ther.

[38] Zhu Y, Huang S, Chen S (2021). SOX2 promotes chemoresistance, cancer stem cells properties, and epithelial-mesenchymal transition by beta-catenin and Beclin1/autophagy signaling in colorectal cancer. Cell Death Dis.

[39] Mirzaei S, Paskeh M, Entezari M (2022). SOX2 function in cancers: Association with growth, invasion, stemness and therapy response. Biomed Pharmacother.

[40] Chen Y, Zhao H, Liang W (2022). Autophagy regulates the cancer stem cell phenotype of head and neck squamous cell carcinoma through the noncanonical FOXO3/SOX2 axis. Oncogene.

[41] Chang TY, Lan KC, Chiu CY, Sheu ML, Liu SH (2022). ANGPTL1 attenuates cancer migration, invasion, and stemness through regulating FOXO3a-mediated SOX2 expression in colorectal cancer. Clin Sci (Lond).

